# Editorial: Exploring innovative experimental paradigms and methods in psychology

**DOI:** 10.3389/fpsyg.2026.1799906

**Published:** 2026-04-01

**Authors:** Olga Daneyko, Valter Prpic, Daniele Zavagno

**Affiliations:** 1 Sheffield Institute of Social Sciences, Sheffield Hallam University, Sheffield, United Kingdom; 2 Department of Theoretical and Applied Sciences, eCampus University, Novedrate, Italy; 3 Department of Psychology, University of Milano-Bicocca, Milan, Italy

**Keywords:** experimental psychology, multisensory processing, new paradigms, risk evaluation, social perception, virtual reality, visual crowding

Experimental psychology, though a well-established field with a rich history spanning over a century, continues to evolve rapidly. In recent years researchers have developed novel methods that allow the study of human cognition, perception, and behavior in ways that are both precise and ecologically valid. This Research Topic presents five innovative studies, each reflecting the creativity and rigor of the scientists behind them. Collectively they illustrate how technological and methodological innovation is reshaping the field.

Liu and Enns have developed an action-observation methodology that enables detailed exploration of social perception. Their innovative approach allows observers to infer attentional states, intentions, and social cues from subtle gestures and eye movements. By revealing how social context and observer perspective modulate perception, and uncovering patterns of coordination in collaborative interactions, their work brings laboratory research closer to real-world social behavior. This paradigm exemplifies how carefully designed methods can open new windows into understanding human social cognition.

Bonny et al. have made a significant contribution to risk perception research by creating a video library of simulated building fires. Through systematic manipulation of fire growth, intensity, smoke opacity, room layout, and viewpoint, their study reveals how environmental and perceptual factors shape human risk evaluation. This method allows safe, controlled investigation of scenarios that would be impossible to study in real life. The team's work not only advances the science of risk perception but also lays a foundation for future immersive virtual reality applications.

In the study of emotional learning, Lucifora et al. have developed PanicRoom, a virtual reality paradigm for Pavlovian fear conditioning. Their VR-based approach enables safe, immersive exposure to fear-inducing stimuli paired with conditioned cues, while behavioral and physiological measures capture fear acquisition and extinction. By overcoming the limitations of traditional shock-based paradigms, the authors provide a standardized and ecologically valid platform for studying fear, anxiety, and related processes. Their ingenuity demonstrates how technology can transform experimental paradigms.

Tanriverdi and Cornelissen introduced innovative methods for rapid assessment of peripheral visual crowding, a phenomenon where object recognition is impaired in cluttered environments. Using eye movement-based serial search and 6AFC paradigms, they significantly reduced testing time while maintaining accuracy. Their work exemplifies how methodological refinement can improve both efficiency and precision, with promising applications for clinical diagnostics in visual and neurological disorders.

Prpic et al. explore the role of auditory cues in drivers' speed perception, highlighting the challenges posed by electric vehicles with reduced engine sounds. Their proposed multi-method approach, combining lab experiments, simulators, and field studies demonstrates how careful experimental design can uncover multisensory contributions to perception and decision-making. The authors' insights have practical implications for transportation safety and illustrate how applied experimental psychology can inform real-world challenges.

Across these five studies a clear theme emerges: innovation in experimental methods is driven by the ingenuity and vision of researchers who combine a rigorous scientific approach with technological tools. Whether through action-observation, video simulations, virtual reality, adaptive psychophysics, or multisensory approaches, these paradigms enable precise measurement while capturing the complexity of real-world behavior. By showcasing the people behind these methods, we recognize that the advancement of experimental psychology relies not only on technology but also on scientists' creativity, insight, and perseverance.

